# How Extended Is Wernicke's Area? Meta-Analytic Connectivity Study of BA20 and Integrative Proposal

**DOI:** 10.1155/2016/4962562

**Published:** 2016-02-23

**Authors:** Alfredo Ardila, Byron Bernal, Monica Rosselli

**Affiliations:** ^1^Department of Communication Sciences and Disorders, Florida International University, Miami, FL 33199, USA; ^2^Radiology Department and Research Institute, Miami Children's Hospital, Miami, FL 33155, USA; ^3^Department of Psychology, Florida Atlantic University, Davie, FL 33144, USA

## Abstract

Understanding the functions of different brain areas has represented a major endeavor of contemporary neurosciences. The purpose of this paper was to pinpoint the connectivity of Brodmann area 20 (BA20) (inferior temporal gyrus, fusiform gyrus) in language tasks. A meta-analysis was conducted to assess the language network in which BA20 is involved. The DataBase of Brainmap was used; 11 papers corresponding to 12 experimental conditions with a total of 207 subjects were included in this analysis. Our results demonstrated seven clusters of activation including other temporal lobe areas (BA3, BA21), the insula, and the prefrontal cortex; minor clusters in the cingulate gyrus and the occipital lobe were observed; however, the volumes of all the activation clusters were small. Our results suggest that regardless of BA20 having certain participation in language processes it cannot be considered as a core language processing area (Wernicke's area); nonetheless, it could be regarded as kind of language processing marginal area, participating in “extended Wernicke's area” or simply “Wernicke's system.” It is suggested that “core Wernicke's area” roughly corresponds to BA21, BA22, BA41, and BA42, while a “language associations area” roughly corresponds to BA20, BA37, BA38, BA39, and BA40 (“extended Wernicke's area” or “Wernicke's system”).

## 1. Introduction

The exact location and extension of Wernicke's area have been inconsistent and polemic [1]. Conceptually, it is easy to define Wernicke's area which corresponds to the language auditory processing area in the left hemisphere [2–4]. Thus, the controversial and unanswered question becomes the following: which are the borders of the auditory language processing area in the left hemisphere?

Since Wernicke [5] the auditory language processing area (later known as “Wernicke's area”) has been generally, but not always, equated with the first temporal gyrus of the left hemisphere. Similarly, when Dejerine [6] referred to the “*language zone*” (or “*language area*”), he included (in addition to Broca's area involved in language production and the angular gyrus participating in written language) the first temporal gyrus (referred to as “Wernicke's area”) related to language understanding. During the following decades, the idea of the so-called language area (roughly corresponding to the peri-Sylvian area of the left hemisphere) was solidly integrated into the aphasia literature (e.g., [3, 7–10]). Most authors included in the temporal segment of the language area (Wernicke's area) the first and sometimes also the second temporal gyrus (usually its posterior section) in general corresponding to Brodmann areas 21 and 22 (BA21 and BA22). Sometimes, the angular and the supramarginal gyri have also been included (e.g., [11]).

Recent research using contemporary neuroimaging techniques (e.g., PET and fMRI) has reanalyzed the exact localization of the language understanding area in the temporal lobe, attempting to pinpoint the functional anatomy of language (e.g., [12–14]). Several proposals have been presented.

Ferstl et al. [15] conducted a meta-analysis of 23 neuroimaging studies on text comprehension directed to confirm the extension of the brain network involved in processing language in context. It was found that independent of the baseline the anterior temporal lobes were bilaterally active. In addition, processing of coherent compared with incoherent text engaged the left dorsomedial prefrontal cortex and the posterior cingulate cortex. Right hemisphere activations were seen most notably in the analysis of contrasts testing specific subprocesses, such as metaphor comprehension. These results suggest that when language comprehension is processed in a context, it is associated with an extensive brain activation network, involving not just the left, but also the right hemisphere.

Démonet et al. [16] used Positron Emission Technology (PET) to analyze brain activation during phonological and lexical semantic processing; they found the first one associated with activation of the left superior temporal gyrus, whereas the latter was linked to activity in the left middle and inferior temporal gyri. These findings are congruent with the aphasia literature, which demonstrates that the superior temporal gyrus damage results in phoneme discrimination defects, whereas lexical impairments are found in cases of middle temporal gyrus pathology, and semantic defects are usually observed in cases of posterior inferior temporal-occipital damage (e.g., [4, 17]).

DeWitt and Rauschecker [12] have proposed that “Wernicke's area” may be better construed as two cortical modules, an auditory word-form area in the auditory ventral stream and an “inner speech area” in the auditory dorsal stream, thus emphasizing the heterogeneity of this language processing area. Dronkers et al. [13] pointed out that traditional language areas, such as Wernicke's area, may serve somewhat different functions than originally described; they suggest that the analysis of more specific deficits and their anatomical correlates can lead to improved mapping of language functions in the brain.

Saygin et al. [18] assessed the relationship between verbal and nonverbal auditory processing by examining the ability of 30 left hemisphere-damaged aphasic patients to match environmental sounds and linguistic phrases to corresponding pictures. Lesion overlay analysis indicated that damage to posterior regions in the left middle and superior temporal gyri and to the inferior parietal lobe was a predictor of deficits in processing for both speech and environmental sounds. The lesion mapping revealed a posterior superior temporal region (Wernicke's area) as being differentially more important for processing nonverbal sounds compared with verbal sounds, suggesting that language shares neural resources with those used for processing information in other domains. Congruent with this observation, Wise et al. [19] using PET identified two anatomically separable, functional subsystems in the left temporal cortex. One part, directed along the supratemporal cortical plane, responded to both nonspeech and speech sounds, including the sound of the speaker's own voice. The second, more lateral and ventral part lay in the posterior left superior temporal sulcus, a region that responded to an external source of speech.

Noteworthy, departing from fMRI studies Binder et al. [20] refer to the existence of left hemisphere temporoparietal language areas outside the traditional “Wernicke area,” namely, in the middle temporal, inferior temporal, fusiform, and angular gyri; it means kind of “extended Wernicke's area.” This implies that, in addition to classical Wernicke's area (first and second temporal gyrus) involved in auditory language processing, there are also some adjacent brain areas, such as the inferior temporal gyrus and the angular gyrus, participating in language processing.

Different recent functional studies have suggested the involvement of BA20 (inferior temporal gyrus and anterior part of the fusiform gyrus) in language processing (see [21]), including lexicosemantic processing [22, 23], metaphor comprehension [24], language comprehension and production [25], and selective attention to speech [26]. BA20 atrophy, on the other hand, has been observed in semantic dementia [27], supporting its involvement in semantic language understanding.

In previous meta-analytic connectivity studies, we illustrated that the left fusiform gyrus (BA37), the left temporal pole (BA38), and the angular gyrus (BA39) were clearly involved in the language processing [28–30]. In this paper, the potential participation of BA20 in language is analyzed. BA20 is bounded medially by BA36 (ectorhinal area), laterally by BA21 (middle temporal gyrus), rostrally by BA38 (temporal pole), and caudally by BA37 (posterior inferior temporal gyrus, fusiform gyrus). This analysis has the purpose of further pinpointing the temporal areas involved in language processing.

Currently, there are several techniques that can potentially demonstrate brain circuitries or networks. These techniques are grouped under the term “brain connectivity." Recently, a new alternative to study brain connectivity has been proposed by Robinson et al. [31] known as* Meta-Analytic Connectivity Modeling *or MACM. MACM is based on automatic meta-analysis done by pooling coactivation patterns. The technique takes advantage of the Brainmap.org's repository of functional MRI studies and of a special software (Sleuth) provided by the same group, to find, filter, organize, plot, and export the peaks coordinates for further statistical analysis of its results. Sleuth provides a list of foci, in Talairach or MNI coordinates, each one representing the center of mass of a cluster of activation. The method takes the region of interest (e.g., BA20), makes it the independent variable, and interrogates the database for studies showing activation of the chosen target. The query is easily filtered with different conditions (such as age, normal versus patients, type of paradigm, and domain of cognition). By pooling the data with these conditions the tool provides a universe of coactivations that can be statistically analyzed for significant commonality. As a final step, Activation Likelihood Estimation (ALE) [32, 33] is performed utilizing GingerALE, another software also provided by Brainmap, assessing the probability of an event to occur at voxel level across the studies. Areas of coactivation will show a network related to the function and domains selected as filter criteria. It is assumed that if two or more areas are activated within the same task, they are interconnected and participate in a single network.

The present study was aimed at demonstrating the networks in which BA20 is involved during the performance of different language tasks using Meta-Analytic Connectivity Modeling (MACM). Simultaneous activation of two or more areas when performing a particular task indicates that those areas may be interconnected and consequently belong to a unified brain network. Coactivation however does not allow conclusion about the direction and sequence of activation.

## 2. Materials and Methods

The DataBase of Brainmap [21] was accessed utilizing Sleuth 2.2 on July 20, 2015. Sleuth is the software provided by Brainmap to query its database. The meta-analysis intends to assess the network of coactivations in which BA20 is involved.

The search conditions were as follows: (1) studies reporting BA20 activation; (2) using fMRI; (3) context: normal subjects; (4) activations: activation only; (5) handedness: right-handed subjects; (6) age 18–60 years; (7) domain: cognition, subtype: language; (8) language: English.

ALE meta-analysis was then performed utilizing GingerALE. ALE maps were thresholded at *p* < 0.01 for multiple comparisons and false discovery rate. Only clusters of 200 or more cubic mm were accepted as valid clusters. ALE results were overlaid onto an anatomical template suitable for MNI coordinates, also provided by BrainMap.org. For this purpose we utilized the Multi-Image Analysis GUI (Mango) [34]. Mosaics of 5 × 6 insets of transversal fusion images were generated utilizing a plugin of the same tool, selecting every other image, starting on image number 10, and exported to a 2D-jpg image. A 3D rendition of the brain was also obtained; the left hemisphere lateral view has been chosen for display.

## 3. Results

Eleven papers corresponding to 12 experimental conditions with a total of 207 subjects were selected (subjects participating in two different experiments were counted as two subjects) (Table 1).

**Figure 1:**
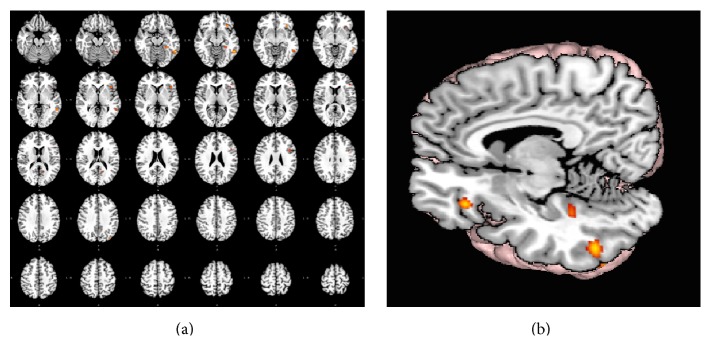
Functional connectivity map of BA20 by Meta-Analytic Connectivity Modeling. (a) Transversal descending cuts of the brain MRI template. Left hemisphere appears on the left side (neurological convention). Clusters of activation are color coded for statistical significance from dark blue (lowest) to red (highest). (b) 3D volumetric rendition of the brain showing activation on the left hemisphere surface. Red color zone identifies BA20. Deep and midline activations are shown.

Table 2 presents the main loci of brain connectivity of BA20 by Meta-Analytic Connectivity Modeling (MACM). Seven different clusters of activation were found, all related to the left hemisphere (Figure 1).

Regardless of the BA20 activation during diverse linguistic tasks, the volumes of the clusters were small (see “volume” in Table 2 and Figure 1). The first cluster includes the left temporal lobe, BA20 and BA21, whereas Cluster #2 was located at the insula (BA13) and the prefrontal BA46. Cluster #3 involved the inferior frontal lobe (BA47) and Cluster #4 was situated in the left inferior temporal lobe (BA37). Cluster #5 was again situated in the left prefrontal cortex (BA9). The last two clusters involved the cingulate gyrus (BA30) (Cluster #6) and the occipital gyrus (BA19) (Cluster #7).

Indeed there were only few activated areas outside the temporal lobe: insula (BA13), prefrontal cortex (BA46 and BA9), cingulate gyrus (BA30), and the occipital lobe (BA19). Yet in all the cases the level of activation was modest; and all the clusters were situated in the left hemisphere.

## 4. Discussion

Regardless of the diverse limitations that can be pointed to the present study (specific characteristics of the sample, implicit limitations of the method that was used, inclusion of language as a whole without distinguishing different language abilities, modality of the stimuli presentation, considering subjects participating in two different experiments as two different subjects, etc.), current meta-analysis illustrates that BA20 has certain, albeit limited, participation in language processes. It is mainly connected with other areas of the temporal lobe, the insula, and the prefrontal cortex. Minor interconnections with the cingulate gyrus and the occipital lobe were also observed.

Retaking the initial question of this paper (“how extended Wernicke's area is?”), our results suggest that, regardless of the fact that BA20 participates in language processes, in no way can it be considered as a core language processing area (Wernicke's area). Hence, it could be interpreted as a kind of language processing marginal area. A similar secondary role in language processing has been suggested for other temporal (BA37 and BA38) [28, 29] and parietal (BA39) [30] areas.

To further pinpoint the extension of Wernicke's area, the potential participation of two additional retrorolandic areas in language should be considered: supramarginal gyrus (BA40) and primary auditory cortex (BA41 and BA42).

The involvement of BA40 (supramarginal gyrus) in language processing is supported by diverse previous reports. Damage in BA40 relates to conduction aphasia [35–37]; and contemporary neuroimaging studies support BA40 involvement in several language functions: attention to phonological relations [38], semantic processing [30], verbal creativity [39], writing [40], phonological processing and verbal working memory [41], and language production [42]. Some authors have even suggested that BA40 should be considered as a part of Wernicke's area [11].

Damage in BA41 and BA42 (primary auditory cortex) on the other hand is associated with “pure word-deafness” (sometimes referred to as “auditory verbal agnosia”) [43–48]; this unusual syndrome is characterized by an inability to understand spoken language with preserved speech production and reading ability [49]. It is usually regarded as a fragment of Wernicke's aphasia [50], and hence it seems reasonable to include BA41 and BA42 as a part of Wernicke's area.

In summary, current information suggests that two posterior language areas can be distinguished: core Wernicke's area (language auditory processing area in the left hemisphere) including not only BA22 and BA21 (as usually accepted), but also BA41 and BA42; a “language associations area” roughly corresponding to BA20, BA37, BA38, BA39, and BA40 (“extended Wernicke's area” or “Wernicke's system”).

## Figures and Tables

**Table 1:** Studies of language paradigms included in the meta-analysis (11 papers, 207 subjects, 12 experiments, and 174 foci).

Publication	Paradigm	*n*	Foci
Devlin et al., 2003	Semantic + phonologic − rest	12	26
Binder et al., 2003	Word > nonwords	26	26
Shapiro et al., 2006	Nouns > verbs	10	3
Katzir et al., 2005	Nonblend > plus-minus	12	12
Assaf et al., 2006	Correct recall	18	15
Wang et al., 2004	Naming > letter strings	12	7
Damasio et al., 2001	Word retrieval − picture control	20	7
	Action words − picture control	20	6
Longe et al., 2007	Verbs + nouns versus baseline	12	8
Binder et al., 2005	Naming − written words	24	32
Sabsevitz et al., 2005	Concrete − abstract nouns	28	26
Bedny et al., 2006	Word comprehension	13	6

**Table 2:** Main loci of brain connectivity of BA20 in language tasks by Meta-Analytic Connectivity Modeling (MACM).

Region (BA)	*x*	*y*	*z*	ALE	Volume (mm^3^)
Cluster #1					
L inferior temporal gyrus (20)	−58	−52	−14	0.0226	1,880
L middle temporal gyrus (21)	−58	−48	−4	0.0197	
Cluster #2					
L insula (13)	−44	24	2	0.0184	968
L frontal lobe (46)	−40	32	8	0.0143	
Cluster #3					
L interior frontal lobe (47)	−34	30	−10	0.0221	584
Cluster #4					
L inferior temporal lobe (37)	−32	−38	−14	0.0180	528
Cluster #5					
L frontal lobe (9)	−44	8	28	0.0161	432
Cluster #6					
L cingulate gyrus (30)	−12	−56	16	0.0179	248
Cluster #7					
L superior occipital gyrus (19)	−38	−80	36	0.0180	208
